# Theta Frequency Background Tunes Transmission but Not Summation of Spiking Responses

**DOI:** 10.1371/journal.pone.0055607

**Published:** 2013-01-31

**Authors:** Dhanya Parameshwaran, Upinder S. Bhalla

**Affiliations:** National Centre for Biological Sciences, TIFR, Bangalore, India; University of Alberta, Canada

## Abstract

Hippocampal neurons are known to fire as a function of frequency and phase of spontaneous network rhythms, associated with the animal's behaviour. This dependence is believed to give rise to precise rate and temporal codes. However, it is not well understood how these periodic membrane potential fluctuations affect the integration of synaptic inputs. Here we used sinusoidal current injection to the soma of CA1 pyramidal neurons in the rat brain slice to simulate background oscillations in the physiologically relevant theta and gamma frequency range. We used a detailed compartmental model to show that somatic current injection gave comparable results to more physiological synaptically driven theta rhythms incorporating excitatory input in the dendrites, and inhibitory input near the soma. We systematically varied the phase of synaptic inputs with respect to this background, and recorded changes in response and summation properties of CA1 neurons using whole-cell patch recordings. The response of the cell was dependent on both the phase of synaptic inputs and frequency of the background input. The probability of the cell spiking for a given synaptic input was up to 40% greater during the depolarized phases between 30–135 degrees of theta frequency current injection. Summation gain on the other hand, was not affected either by the background frequency or the phasic afferent inputs. This flat summation gain, coupled with the enhanced spiking probability during depolarized phases of the theta cycle, resulted in enhanced transmission of summed inputs during the same phase window of 30–135 degrees. Overall, our study suggests that although oscillations provide windows of opportunity to selectively boost transmission and EPSP size, summation of synaptic inputs remains unaffected during membrane oscillations.

## Introduction

Oscillations in the brain have been suggested to provide single neurons with a temporal reference to network activity. The phase of spiking with respect to these oscillations is believed to play a role in information coding of the stimulus features [1]–[4]. The hippocampus generates rhythmic activity at unique behaviourally relevant frequency bands [5]. The output firing of hippocampal cells is thus a combined response to the internal state governed by the network oscillations and external inputs it receives through the afferent fibres [6].

Hippocampal rhythms are typically in the theta (4–12 Hz) and gamma (30–100 Hz) frequencies. Both theta and gamma oscillations have been shown to be emergent properties intrinsic to the hippocampal network [7]–[9]. Theta rhythm is typically seen during REM sleep, spatial tasks and during learning tasks [1], [10]–[12]. Gamma rhythms are seen in the hippocampus during sensory stimulation, working memory maintenance and attention based tasks [13]–[15]. Gamma rhythms can be classified into distinct fast and slow frequency components routing inputs to CA1 from the medial entorhinal cortex and the CA3 region [16]. Lower frequency network oscillations are used for high level temporal interactions with distant regions whereas higher frequency gamma rhythms are implicated in more local computations [17], [18]. These rhythms are not exclusive, and gamma activity is frequently nested within theta waves [19]–[21].

Here we ask the question – do membrane oscillations tune synaptic summation in hippocampal CA1 neurons? Studies on single neuron computation have shown that stimulus at the rising phase of theta oscillations can reliably potentiate the cellular response, which can be depotentiated by stimuli at the falling phases [22]–[24]. *In vivo* and *in silico* studies in the cortex have shown that the level of network activity (UP or DOWN states) affects the amplitude and duration of the evoked post-synaptic potential (PSP) but the summation remains linear with asynchronous inputs [25], [26]. The above studies suggest the possibility that oscillations provide windows of opportunity where summation of synaptic inputs is more effective.

Here we measured the postsynaptic and spiking responses of CA1 pyramidal neurons to summed synchronous synaptic input in the presence of sinusoidal current input representative of network oscillations. Our experiments were designed as a physiological implementation of the intracellular environment and readouts during theta rhythm. This allowed us to simplify the oscillatory interference model [4], [27] to a sinusoidal current injection. Since this technique restricts oscillatory changes to a single point and the non-linear dendritic interactions may not be captured [4], we used a detailed computer model to explore a variety of rhythms and combinations of dendritic and somatic input [26].

Together, these experimental and modeling results show that the theta rhythm robustly gates a window for transmission of synaptic input, but linear summation of multiple inputs remains unaffected at all phases.

## Results

In this study, we investigated the role played by rhythmic hippocampal activity represented by sinusoidal current injection, in tuning responses and summation properties of hippocampal neurons to synaptic inputs. The sinusoidal current injection at 4 Hz, 40 Hz and 100 Hz were designed to simulate the fluctuations in membrane voltage during theta and gamma (slow and fast) spontaneous network activity typically seen in CA1 neurons *in vivo* [28]–[30]. We first characterized the responses of single neurons to increasing levels of sinusoidal current injection at each of the three frequencies – 4 Hz, 40 Hz and 100 Hz. We then delivered synaptic inputs through the afferent fibres (Schaffer Collaterals) at different phases of the background sinusoidal input. We used a computer simulation to explore how different rhythm frequencies of synaptic input might affect the summation. Finally, we delivered multiple simultaneous synaptic inputs to study the effect of sinusoidal current injection on both the spiking response and summation properties of CA1 neurons.

### Firing Rate Is Entrained to Background Current Injection

**Figure 1:**
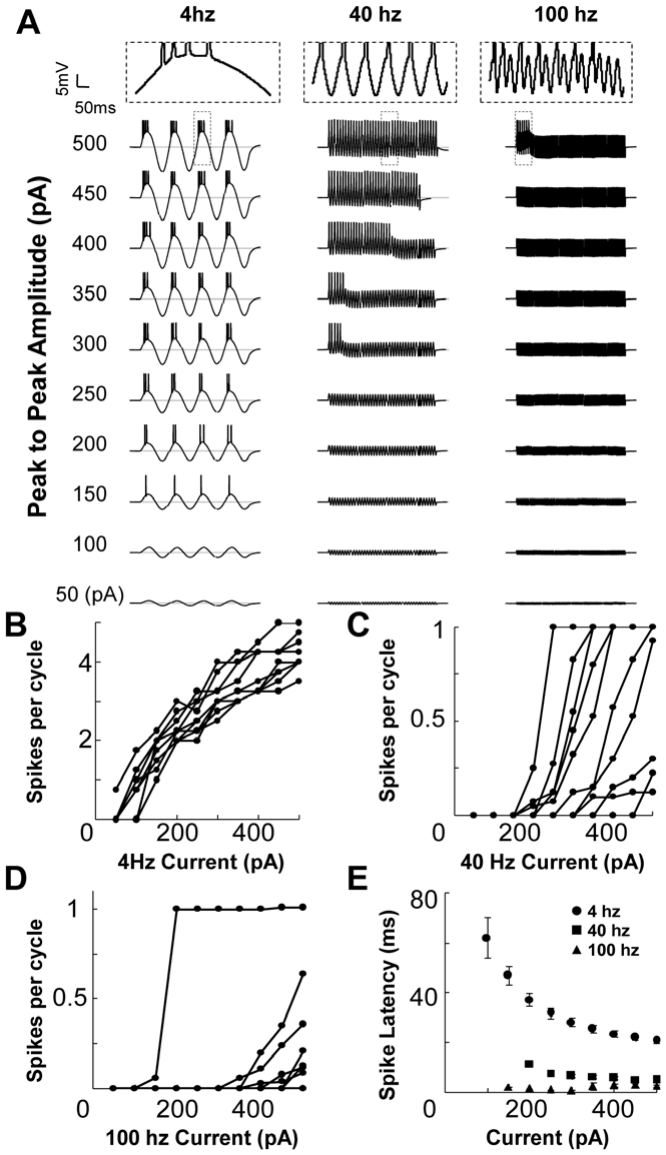
CA1 response entrained by sinusoidal current injection. (**A**) Sine current injection into a CA1 neuron at increasing peak to peak amplitude (y-axis) at 4 Hz, 40 Hz and 100 Hz (left to right). The APs have been clipped at 20 mV. Scale shown in the top left corner. Inset shows expanded views of the dashed box regions. The AP firing is entrained to the frequency of the sine wave especially with 4 Hz injection. Additionally, the cell spikes earlier in the cycle with increasing current amplitude. At 40 Hz, again the spikes are entrained to the frequency of the sine wave, but the cell goes into a quiet state after firing, possibly due to inactivation of active channels. In the 100 Hz case, it is difficult to make the cell fire even at high current amplitudes. (**B–D**) Average spikes per cycle as a function of peak-to-peak amplitude of current injected (N = 11 cells) in the 4, 40 and 100 Hz cases. The firing rate is entrained by the frequency of current injection. The increase in firing rate is sigmoidal in the 40 Hz case. (**E**) The latency of the first spike shows significant phase advancement only with the theta frequency current injection. Error bars represent SE.

In order to investigate the response of the CA1 pyramidal neuron to oscillatory input, we injected sinusoidally modulated current at increasing amplitudes. The firing pattern of CA1 neurons to increasing tonic current injection has been previously studied [31], [32]. We injected increasing amplitudes of sinusoidally modulated current at each of the 3 frequencies to simulate theta, slow gamma and fast gamma rhythms respectively (4 Hz, 40 Hz, and 100 Hz, Figure 1A). This gave us firing rate versus input curves (f-I curves) and firing phase for each of the 3 frequencies. Figures 1B–1D show average spikes per cycle of the sine wave injected, calculated as
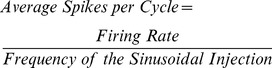
(1)We found that on injecting theta frequency (4 Hz) sinusoidal current the cell reached a maximal firing rate of 20 Hz similar to tonic current injection [31]. The firing rate was entrained by the frequency of the current injection (Figure 1B).

In the case of slow gamma frequency (40 Hz) current injection, the cells showed a non-linear sigmoidal increase in the firing rate response. However at higher current amplitudes the cells were eventually entrained by the stimulus frequency and fired at 40 Hz (Figure 1C). The duration of firing increased with increasing current amplitude. It was difficult to make the cells fire at all with the fast gamma (100 Hz) current injection. This is presumably because depolarization was for too short a time to allow the feedback due to the Na current to build up to an action potential. At very high currents, the cells eventually began to fire (Figures 1A and 1D). These strong stimuli are unlikely to be representative of physiological conditions, and were analyzed here for completeness of the stimulus series. There was considerable variability between the response curves of different cells at higher frequencies of current injection (Figure 1D).

In a behaving animal, the number of spikes fired by a CA1 neuron is known to encode spatial location. As the animal repeatedly runs through the cell's place field its spike timing advances in phase with respect to the theta rhythm [2], [3]. The spike timing and phase thus encode the animal's location. We therefore calculated the timing of action potential (AP) firing from the response curves to the sinusoidal current injection. With increasing current the first AP fired at an advancing phase. However, we found significant phase advancement of the first AP with increasing current amplitudes only in the case of theta frequency (p≪0.05, one-way ANOVA performed for latencies across the various current amplitudes). The spike latency was inversely proportional to the current amplitude (Figures 1E and S1). We did not observe phase advancement with increasing current amplitude in the case of slow and fast gamma frequency current injections.

This characterization was essential since we use sinusoidal current injection in our subsequent experiments to simulate theta and gamma rhythms. Additionally, these experiments allowed us to fix the current amplitude of sinusoidal injection at 60 pA. At this current amplitude the cell did not fire action potentials, and generated oscillations approximating membrane potentials recorded *in vivo* [28], [33]. Additionally, we found that the low-pass filtering properties [32] of CA1 neurons resulted in sparse spiking and negligible tuning in spike timing during gamma frequencies. These results suggest that theta frequency inputs may have stronger phase-dependent effects on spiking responses than gamma frequencies.

### Phasic Response to Synaptic Inputs is a function of the Background Rhythm

**Figure 2:**
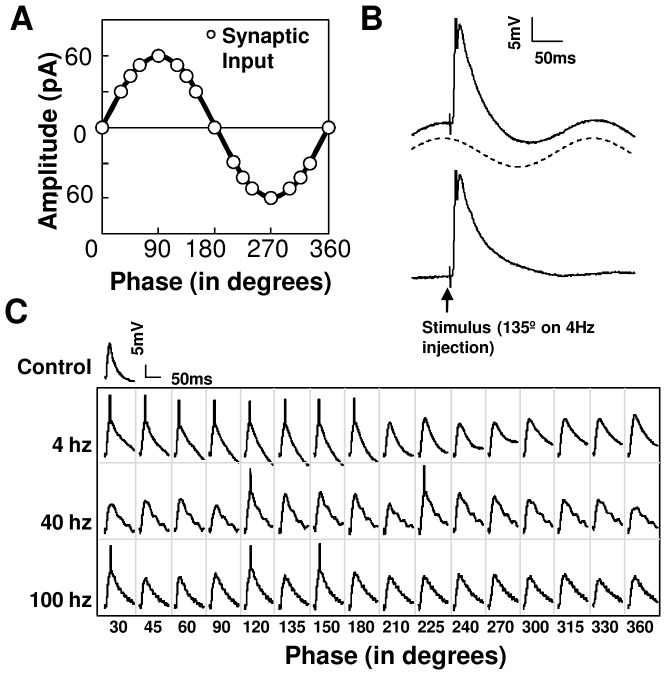
Experimental Design. (**A**) Schematic of stimulus phase relationships. Synaptic inputs were delivered through an electrode to the Schaffer Collaterals at 16 different input phases on the sine-wave background current injection into a CA1 neuron (shown by circles). Thick black line represents the zero mean background input. (**B**) Response readout EPSP area of the non-spiking region gave a measure of the amount of charge injected into in the cell from a single EPSP trace. The APs have been clipped at 20 mV. The latency of the AP was also measured from the stimulus artifact. In the example shown, the afferent input arrives at 135° to the theta frequency current injection. We calculated the positive area up to 200 ms after the stimulus artifact to get the total EPSP area (top panel). We then obtained a best fit sinusoid to the cell's response (dashed line). This fitted response was then subtracted from the EPSP trace to get the subtracted EPSP area (bottom panel). (**C**) Raw traces (each of a single trial) shown for a cell's (#210510s1_c1) response to synaptic inputs combined with sine current injection. At certain phases (4 Hz, 0–180 degrees), the synaptic input results in an AP (trimmed). The response of the cell to just synaptic inputs without current injection was used a control. The response of the cell is dependent both on the frequency of sinusoidal current injection and on the phase of synaptic input.

**Figure 3:**
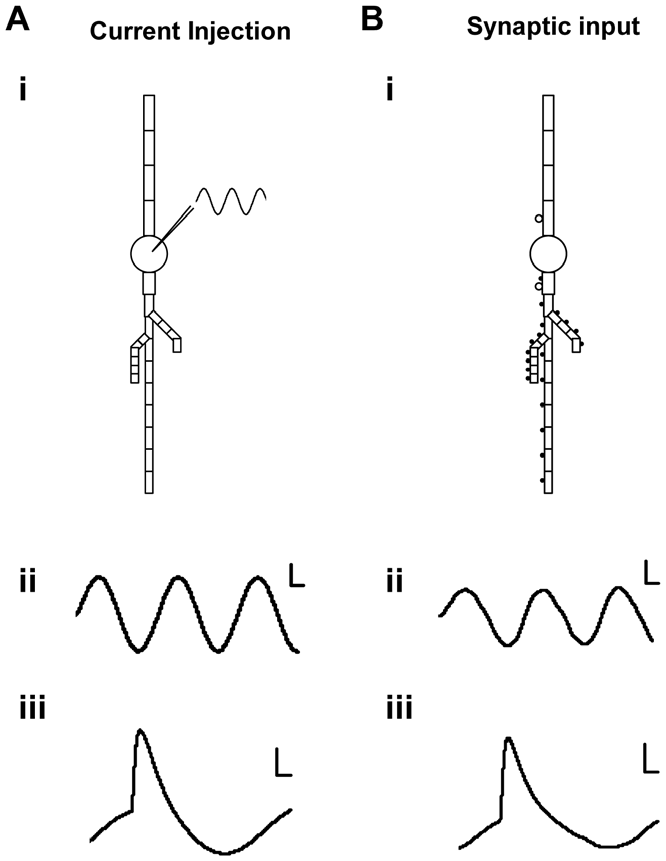
Model Design. CA1 neurons were modelled using 25-compartments with the inclusion of NMDA, AMPA and GABA receptors. Note that in the cell model schematics, the basal dendrites are positioned upward and the apical dendrites downward, consistent with typical recording configurations. (**A**) Theta frequency network activity simulated using sinusoidal current injection. This generated a sinusoidal response at the soma with a peak-to-peak membrane potential of ∼10 mV (sub-panel ii, Scale bars represent 1 mV, 20 ms). Current injection was then given in combination with afferent synaptic input at 16 input phases. Example in sub-panel iii shows afferent inputs converging at an input phase of 90 degrees (Scale bars represent 2 mV, 10 ms). (**B**) Theta frequency network activity simulated using a patterned barrage of excitatory and inhibitory inputs. GABA inputs were clustered on the soma and on the most proximal apical and basal compartments (open circles). Glutamate and NMDA receptors were distributed throughout the apical dendrite including its branches (filled circles). Sub-panel ii shows the resulting sinusoidal theta frequency response measured at the soma (Scale bars represent 1 mV, 20 ms). Afferent synaptic inputs were then overlaid at 16 input phases. Example in sub-panel iii shows afferent inputs converging at an input phase of 90 degrees (Scale bars represent 2 mV, 10 ms). Somatic responses generated using current injection are comparable to those generated using a barrage of synaptic inputs.

We next considered interactions of rhythmic (theta and gamma) activity with excitatory synaptic inputs [34]. To replicate this condition *ex vivo*, we systematically stimulated the hippocampal neurons with afferent synaptic input in combination with sinusoidal current injection (Figure 2). Synaptic input was given at 16 different input phase values (Figure 2A). We hypothesized that responses to afferent synaptic input might be a function of frequency of background input, or of input phase, or both. We used latency of AP firing and EPSP area as our response readouts for spiking output and the driving force (Methods). We then replicated this experiment *in silico* by simulating the inputs to the CA1 neuron to calibrate the current injection responses (Figure 3A) with network activity represented as barrages of excitatory and inhibitory inputs (Figure 3B) (Methods).

**Figure 4:**
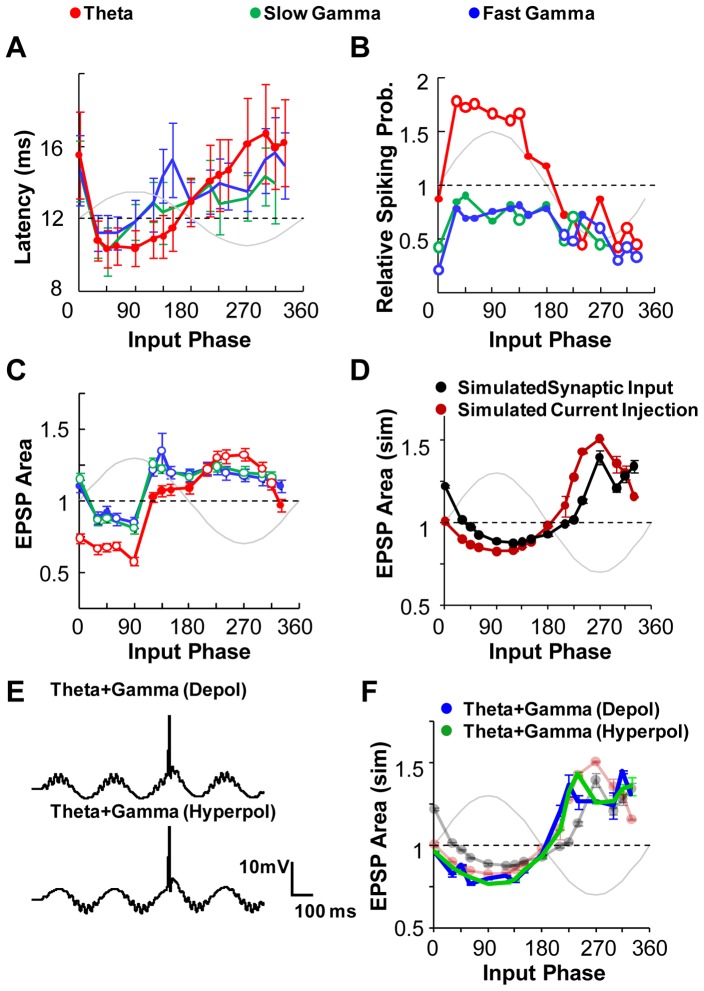
Rhythmic Input Shapes Response to Synaptic Input. Mean response of hippocampal CA1 cells to synaptic input arriving at 16 different phases of the injected sine wave. Sine waves of 3 frequencies were injected (Red: theta; Green: slow gamma, Blue: fast gamma). The dotted line parallel to the x-axis represents the control case with no background current injection and the sinusoidal gray trace shows the somatic current injected at the input phases. Open symbols represent significant data points (in comparison with the control group). Error bars represent SE. (**A**) Latency of spiking is a function of the phase with both theta and gamma frequency background. The cell spikes earlier in the depolarizing phase of sinusoidal current injection. This effect is more pronounced for theta frequency current injection but does not cross the significance criterion of p<0.05. (**B**) The probability of spiking following synaptic input is significantly higher between 30–135 degrees input phase with theta current injection. The probability of spiking is below the control for all values of input phase with slow and fast gamma frequency current injection. Data points that are significantly higher or lower (Binomial Test, p<0.05) than control are represented as open symbols. (**C**) Subtracted EPSP area is a function of both the phase of synaptic input and frequency of current injected. Area is normalized to control responses with no background current. The background sine wave injected is centered on zero current. The best fit sinusoid to the cell's response was subtracted from the EPSP trace. Data points that are significantly higher or lower (Student's t-test, p<0.05) than control are represented as open symbols. (**D**) Somatic current injection and network activity generated using a barrage of excitatory and inhibitory inputs caused similar phasic modulation in sinusoid subtracted EPSP area to afferent inputs. The comparison was done using an *in silico* model of CA3-CA1 network. The phasic modulation is qualitatively similar to responses measured in whole-cell recording experiments. (**E**) Theta-gamma coupled inputs paired with afferent inputs were simulated using the current injection protocol. Two types of input were given – one, with the maximal power of gamma frequency input aligned with the depolarized phase of theta input (above). Two, the maximal power of gamma input coincided with the hyperpolarized phase of theta input (below). (**F**) The phasic tuning of EPSP area with theta-gamma coupled input closely matched the tuning seen with just theta frequency background. The lines in the red and black as same as those shown in Figure 4D.

Does the phase-tuning of synaptic response manifest in spiking output? We measured spiking latency and spiking probability as a function of phase of afferent input (Figures 4A and 4B). We found a strong phase-tuning of spiking probability (p<0.05, Binomial test) in the case of theta frequency background. There was a complementary decrease in spiking latency with a negative covariance with instantaneous background current of −0.93 (p<0.005), again in the case of theta frequency background. In contrast to the theta frequency, slow and fast gamma frequency current injections had a smaller phase-dependence of latency and firing probability. The spiking probability was lower than control for all input phases (Figure 4B).

To further characterize spiking phase dependence, we estimated how spike generation lagged the synaptic input. We defined Phase Lag as
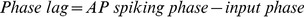
(2)At theta, slow and fast gamma frequency backgrounds, phase lag remained constant with the input phase [35] (Figure S2). The coefficient of variance of phase lag was comparable across the three frequency backgrounds (coefficient of variance of 0.12, 0.11, 0.10 for 4, 40 and 100 Hz backgrounds). We also analysed the effect of background input on the spike timing precision. Jitter in spike timing has been shown to be low for synchronous synaptic inputs like the afferent inputs used in this study [36]. We did not find any significant phasic modulation of jitter (measured as standard deviation in spike latency across trials, Student's two-sample t-test, p>0.05) with either frequency of background or input phase.

We measured how EPSP area varied with the input phase, and with the frequency of current injection. We used two cycles of the voltage trace after the end of the EPSP to obtain a best fit sinusoid to the cell's response. This fitted response was then subtracted from the EPSP trace (Figure 2B). We found that both the amplitude and duration of the EPSP were functions of the input phase. EPSP area was lower at the depolarising phases (30–90 degrees) and higher for hyperpolarising phases (210–315 degrees) with all three background frequencies (Figure 4C). Specifically, the EPSP area differed significantly from control for all input phases except 120–180 degrees with theta frequency background (Student's two-sample t-test, p<0.05). EPSP area was negatively correlated with the instantaneous amplitude of background current injected (Pearson's correlation coefficients, r = −0.77, p<0.0005 for theta, r = −0.54, p = 0.066 for slow gamma and r = −0.46, p = 0.072 for fast gamma frequency current injections). An earlier extracellular study in CA1 neurons reports fEPSP slope dependence on theta rhythm input phase that is consistent with our intracellular readouts shown in Figure 4 [37]. This is an important validation for the use of sinusoidal current injection as representative of *in vivo* oscillations.

Together, these results suggest that the response of the cell, as measured by spiking probability and by EPSP, appears to be tuned by the input phase with respect to background rhythmic activity. Further, the large changes (∼50%) in EPSP size which we see with background rhythmic input suggest that driving force may not be the only reason for phase tuning.

### Sinusoidal Current Injection is a good approximation for rhythmic network activity

Several single neuron studies suggest the role of dendritic activity in introducing non-linearities in the somatic response. Such studies suggest that these nonlinearities may not be captured using a simple current injection protocol [4]. To examine this, we built a 25-compartment model of a CA1 pyramidal neuron embedded in the CA3-CA1 network to simulate and compare the responses with the current injection and synaptic input protocols for theta frequency (Methods, Figure 3). Briefly, the synaptic input protocol utilized excitatory (GluR and NMDAR) synaptic input in the dendrites, and inhibitory (GABA-R) input at and near the soma. The excitatory and inhibitory inputs were delivered 180 degrees out of phase with each other as suggested by the somato-dendritic interference model [4], [27]. We ran both simulation protocols for 5 trials and compared the phasic change in average area under curves (Figure 4D). We found that network activity simulated using synaptic inputs causes a similar phasic modulation of area under the curve as current injection (Pearson's correlation coefficient r = 0.79, p<0.0005). However, in the synaptic input simulation we found a phase delay of ∼21 degrees. This phase shift was caused due to the capacitive elements in the model which were compensated for in the whole-cell recordings. We also ran simulations with 8 and 12 Hz frequency backgrounds using both the current injection and synaptic input protocols. Our results with 8 and 12 Hz theta rhythms gave similar phase tuning to the 4 Hz. However, as expected, phase shift between the current injection and synaptic input cases, increased with the increase in the input frequency (Figure S3, phase shift at 8 Hz = ∼40 degrees, 12 Hz = ∼70 degrees).

We then compared the simulation results (with 4 Hz theta) with that observed in the whole-cell recordings. We found a strong correlation between the *ex vivo* and *in silico* current injection experiments (r = 0.75, p<0.005). The phasic relationship of lower EPSP area in the depolarizing phases and higher EPSP area in the hyperpolarizing phases was consistent across both the simulations and the recordings (r = −0.77, p<0.0005 for whole-cell recording, r = −0.96, p<0.0005 for simulated current injection and r = −0.78, p<0.0005 for simulated synaptic input case).

We then used the simulations to investigate the role of active channels in reducing EPSP area during depolarized phases and enhancing EPSP area in the hyperpolarized phases. As detailed in the methods section, we convolved the EPSC with the time-profile of the inverse of the net conductance. We repeated this calculation for all stimulus phases. We carried out these calculations first with all somatic voltage-gated ion channels, and second with only the K-channels. First, we observe that the estimated modulation of EPSP area due to channel conductances is similar in phase to the observed modulation of EPSP area in experiments and in simulations (r = 0.73, p<0.001). However, the amplitude of this modulation is smaller than in the experiments and simulations by a factor of about 2. Second, we found that K-channels account for almost all of the modulation of EPSP area (Figure S3) (correlation between K-channel and all channel contribution r = 0.97, p<0.00001). These results suggest in addition to driving force, the modulation of K-channel conductances may also contribute to the phasic modulation of EPSP area.

We conclude that the summation of Schaffer Collateral volley input with sinusoidal current injection is a reasonable approximation to the summation of SC input with *ex vivo* theta rhythms generated by periodic excitatory and inhibitory synaptic input.

### Theta-gamma coupled input causes similar response tuning as theta frequency

Does gamma frequency input coupled with theta frequency affect the phase tuning? This possibility is suggested by the observation that gamma rhythms in the hippocampus are frequently observed riding on the theta rhythms [21]. Cross-frequency coupling between theta and gamma rhythms has been proposed to assist in the encoding and retrieval of short-term memory [10], [19].

We simulated cross-frequency coupled background inputs in the 25-compartment CA1 neuron model (Methods, Figure 4E). The gamma frequency (2–3 mV) was co-modulated with theta frequency (5–7 mV). We tested 2 cases – with gamma frequency having maximal power at either depolarized theta (90 degrees) or at hyperpolarized theta (270 degrees) [20], [21]. We recorded the phasic change in average area under curves with both the current injection and synaptic input protocols. Interestingly, we found that responses with the cross-frequency input current injection closely matched our responses with just theta frequency input (Pearson's correlation coefficient r = 0.9, p<10^−5^ for gamma on theta depolarizing; r = 0.93, p<10^−6^ for gamma on theta hyperpolarizing) (Figure 4F).

We also compared responses using synaptic input to drive the coupled theta and gamma rhythms. Here we found that the response tuning with gamma on theta depolarizing case correlated positively with the response tuning seen with just theta background (r = 0.5, p<0.052 and Figure S4). However the gamma on theta hyperpolarizing did not exhibit strong tuning (correlation with only theta background r = −0.17, p<0. 51 and Figure S3). We suggest that the mechanisms for eliciting these coupled rhythms need further detailing in order to establish the physiological relevance of this observation. Overall, the cross-frequency coupling simulations provide additional support for the idea that phasic tuning of CA1 neurons is largely influenced by the slower theta component, and that the gamma component has little effect.

### Summation of Synaptic Inputs is sub-linear

In order to establish baseline summation properties of afferent inputs in our preparation, we next asked how synaptic inputs summed in the absence of background current injection. We positioned an array of four stimulating electrodes on the Schaffer collaterals (SC) of rat hippocampal brain slices. We stimulated these electrodes in different combinations, and measured how their responses summed (Methods).

Previous studies have shown that the distribution of synapses receiving input on the dendrite decide the mode of integration in CA1 neurons [36], [38]. Summations investigated in this study represented spatially distributed, synchronous synaptic stimuli (Methods S1 and Figure S5).

**Figure 5:**
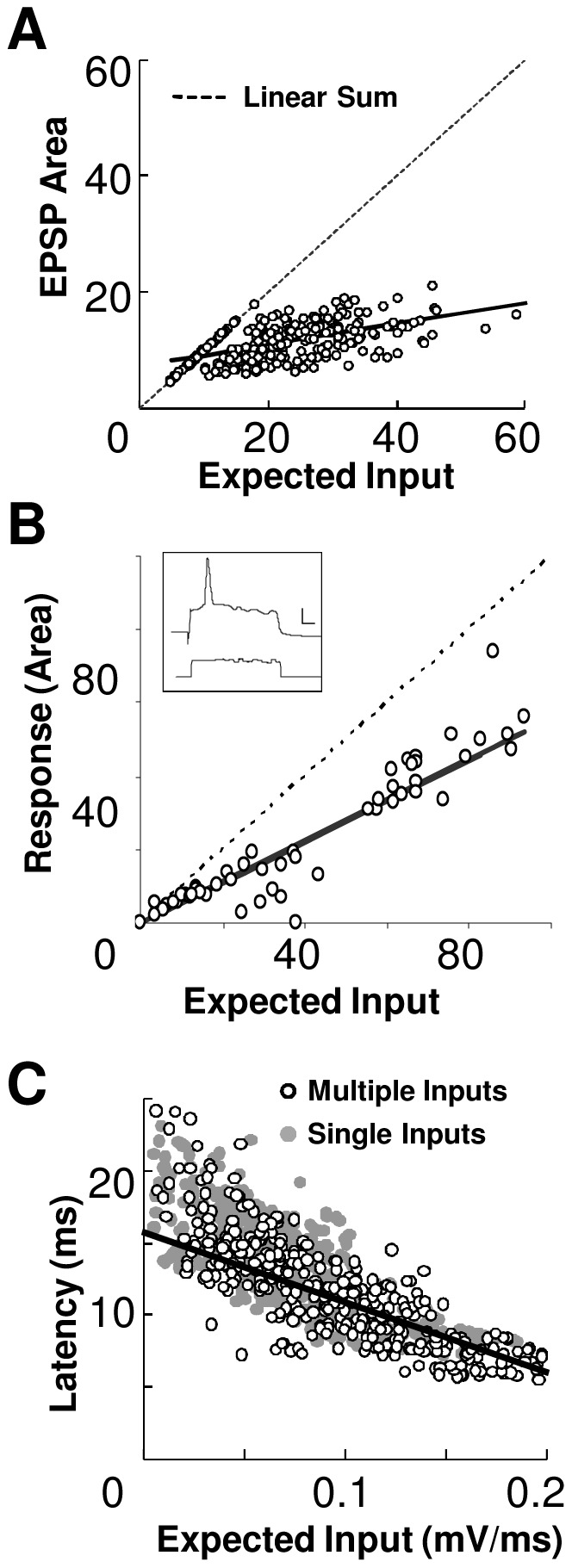
Summation of Synaptic Inputs. Whole-cell patch recordings from CA1 neurons to multiple synaptic inputs without background current injection. In all panels the solid line represents the linear regression fit and the dashed line represents a slope of 1. (**A**) Responses of CA1 neurons to multiple inputs using area under EPSP as readout, plotted against sum of responses to individual inputs. Data is plotted for 15 stimulus combinations on 4 electrodes (N = 14 cells). (**B**) Responses of CA1 neurons (N = 11 cells) to multiple inputs in the presence of background somatic current injection using area under EPSP as a readout. Inset shows response (above) from a cell (280509_c3) to Gaussian noise background current injected (below). The synaptic stimuli were given at the onset of the background current. Scale bar for current injected (X-axis 5 ms, Y-axis 100 pA). Scale bar for voltage response (Y-axis 10 mV). (**C**) Latency to fire first AP is negatively correlated with total input (N = 14 cells, averaged over 10 trials). Expected input was calculated as the sum of EPSP slopes of single inputs.

We used whole-cell patch recordings readouts to record single neuron integration of synaptic inputs without any additional somatic current injection. We used 3 readout parameters - number of action potentials fired, area under EPSP and latency of first AP and compared actual response with expected response. The expected response was calculated as a linear sum of individual input responses. A slope of actual response to expected response of 1 represent linear summation, <1 represents sub-linear summation and >1 represents supra-linear summation. We find that although the response had a good linear fit, with a slope of 0.65 (linear regression fit, R^2^ = 0.851) the summation was sub-linear (Figure 5A).

We found that the number of APs was not a good analog measure of input received by the neuron, because in most cases the CA1 neuron responded with a single AP even for strong, multi-electrode synchronous synaptic stimulation [36]. We further tested if the presence of background neuronal input might linearize the output response as measured by action potentials. We simulated background input using steady as well as noisy current clamp inputs. Here too, the number of action potentials remained a poor readout of summed cellular inputs (Figure 5B). Finally, we looked at the latency of AP firing as a measure of the summed input. As expected, the latency decreased when total input increased. Additionally, the latency of AP firing (time to fire from the stimulus artifact) correlated negatively (Pearson's correlation coefficient −0.52, p<0.01, linear regression fit R^2^ = 0.95) with the total input (Figure 5C). The spike latency never fell below a minimal value of 5.7±1.2 ms (mean ± s.d.).

Overall, the intracellular recordings showed that the summation of neuronal input is sub-linear with a gain of 0.65 encoded in intracellular potential readouts such as EPSP area.

### Rhythmic excitation tunes Transmission of Summed Synaptic Inputs

**Figure 6:**
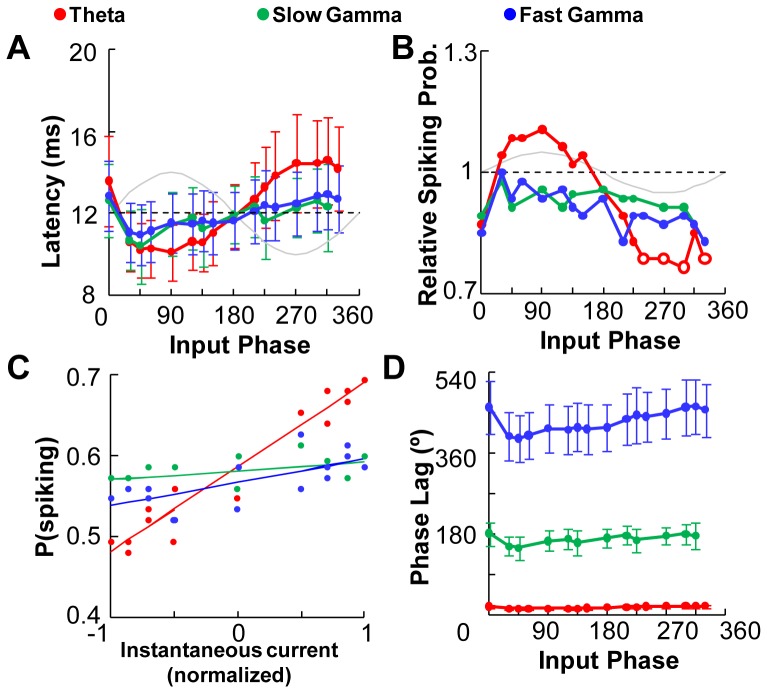
Rhythmic Input Shapes Spike Probability. The dotted line parallel to the x-axis represents the control case with no background current injection and the sinusoidal gray trace shows the somatic current injected at the input phases. Error bars represent SE. (**A**) Latency of evoked APs by summed inputs (N = 12 cells) is also a function of the phase. It is not significantly affected by the summation. (**B**) Probability of spiking is tuned to input phase for summed inputs. The probability dipped significantly between 225–330 degrees only for theta frequency background. In the case of the gamma frequency backgrounds, the probability was below the control (without background current injection) for all values of input phase. Data points that are significantly higher or lower (Binomial Test, p<0.05) than control are represented as open symbols. (**C**) High positive correlation between the probability of spiking and the instantaneous current injected at input phase is seen with only theta frequency background. The lines indicate the best fit found using linear regression (R^2^ of 0.93, 0.18 and 0.46 for theta, slow and fast gamma frequencies respectively). Data shown for only summed synaptic inputs. (**D**) Phase lag remains constant with changing input phase. This relationship remains unaffected on summation for all three background frequencies.

**Figure 7:**
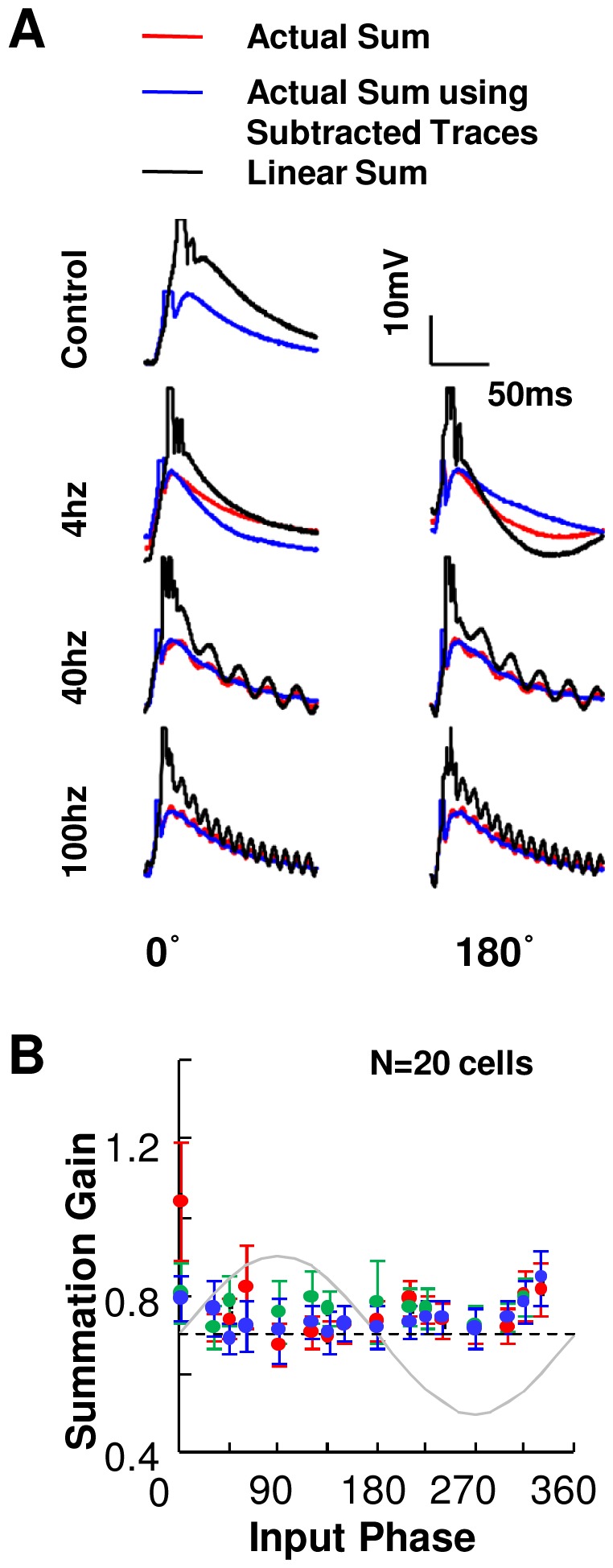
Summation Gain robust to Rhythmic Input Shapes Transmission. (**A**) Example traces (cell # 010410s2_c3) show response to multiple inputs (red) for 2 input phase values of 0 and 180 degrees. Linear sum shown in black, the actual summed response shown in red and the sinusoid subtracted summed responses in blue. (**B**) Summation gain (N = 20 cells) measured as a ratio of sinusoid subtracted EPSP area for multiple inputs to EPSP area for sum of single inputs. On subtracting the best fit sinusoid from the responses, there is no significant phasic modulation with either theta or gamma frequency current injection. Error bars represent SE. The gray trace shows the somatic current injected at the input phases.

Having established baseline summation properties (Figure 5), and the phase-dependence of single-input response to background sinusoidal input (Figures 4), we next combined the two cases. Here we asked if synaptic input summation rules were also modulated by background network activity, represented here by sinusoidal somatic current injection (Figures 6 and 7). This experiment tested several possible outcomes. Specifically, a gain of >0.65 would result in even stronger phase tuning of summed responses; gain of <0.65 would reduce phase dependence, and gain = 0.65 would preserve phase dependence of responses. Phase-selective summation on the other hand would result in a shift in the phase tuning.

The electrode array consisted of 2 electrodes, so that the summation properties could be measured by comparing responses of individual electrodes with the response to combined stimulus. We again used AP timing and EPSP area as the supra-threshold and sub-threshold response readouts. This allowed us to address the role of the background activity in synaptic summation.

As with the single afferent input, the latency of spiking following summed inputs was smallest in the phase window between 45 and 135 degrees for theta-frequency background input but the changes were not significant as compared to the control (Student's t-test p>0.3, Figure 6A, see also Figure S6). This effect also reflected in the phase tuning of the spiking probability (Figure 6B). Spiking probability increased in the phase window between 30–150 degrees, and significantly decreased between 225–330 degrees for the theta frequency background injection (p<0.05, Binomial Test). Thus the summed inputs experienced modulation in both depolarizing and hyperpolarizing phases of theta. There was a high correlation (Pearson's correlation coefficient, r>0.9) between spiking probability and instantaneous background current with theta rhythm, but this correlation was weaker for the two gamma frequencies (Figure 6C). The maximum absolute spiking probability of 0.69 (11% above baseline and 20% above average) was recorded at input phase of 90 degree with theta frequency background current injection. However, spiking probability was nearly flat and consistently below control with both slow and fast gamma frequencies (p>0.05, Binomial Test). We also looked at the relationship between the AP phase lag and precision. Both phase lag (coefficient of variance of phase lag 0.14, 0.14, 0.13 for 4, 40 and 100 Hz backgrounds) and jitter in spike timing remained constant for all input phases and background frequencies (Figure 6D).

We computed a sub-threshold measure of linearity for each phase of synaptic input for theta, slow gamma and fast gamma frequencies of sinusoidal current injection (Figure 7A):
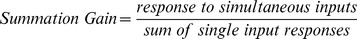
(3)Sub-linear summation in the depolarizing phases and supra-linear summation in the hyperpolarizing phases would result in strong phase tuning of summation gain and vice versa. Uniform summation on the other hand would result in a flat relationship between summation gain and phase.

We subtracted the sinusoidal component from the EPSP traces and measured phasic modulation of summation gain using EPSP area. We found no significant phasic modulation of summation gain with either theta or gamma frequency current injections (Figure 7B). We did not find any significant correlation between the summation gain and instantaneous background current injection (Pearson's correlation coefficients r = −0.17, p = 0.52 for theta, r = 0.22, p = 0.48 for slow gamma and r = −0.38, p = 0.14 for fast gamma frequency current injections). We thus conclude that the summation remained uniformly sub-linear as measured by EPSP area.

Overall, these results support sub-linear and phase-independent summation of synaptic input. This applies both to strong inputs which elicit spiking, and to sub-threshold summation.

## Discussion

Here we studied the role of background rhythmic input in shaping response and summation properties of single neurons. We found that theta but not gamma background oscillations contribute to stimulus information coding in hippocampal neurons. Specifically, we found that the probability of synaptic stimulation to evoke cell firing is higher at the depolarizing phases of theta frequency input. Furthermore, the summation properties of cells remained robustly sub-linear, both with theta and gamma frequency rhythms. Thus, summed inputs behaved with a similar theta phase-dependence as the individual inputs, and spiking responses.

### Computational Implications of Phase Dependent Firing

In many brain areas, the firing rate code is strongly modulated by sensory inputs and the behavior of the animal. It is also known that using additional information about the timing of firing with respect to network activity rhythms may refine encoding, and contribute to behavioral accuracy as seen during phase precession in spatial tasks [2], [27], [39]. This output phase code allows precise prediction of the animal's location from neuronal response. Our results show that phase coding is analog in nature, thus resulting in a gradual modulation of responses with phase. It suggests two possible mechanisms that may result in phase advancement – one, by ramping up the excitatory inputs, thus resulting in an increase the amplitude of the theta rhythm (Figure 1C) and two, modulating the input phase of the afferent inputs. We show that modulations in input phase result in corresponding shifts in the output phase (Figure 4A). Furthermore the slow changes in membrane potential as seen during theta but not gamma frequency oscillations are necessary to facilitate phase coding.

Phase coding has also been observed with gamma oscillations as temporal reference especially in the cortex. Pyramidal neurons in the visual cortex are known to preferentially fire in a small temporal window when the network is engaged in gamma oscillations. Furthermore, cells that do not fire early are not able to fire at all in the cycle [40]. A study by Burchell et. al. has earlier shown that gamma frequency oscillations are shut down by afferent inputs to the same synapses [41]. However, we do not see this effect in our study where our afferent synaptic inputs are independent of the membrane oscillations and we do not activate the GABAergic network with our oscillatory input. Our study showed marginal input phase tuning of responses with both slow and fast gamma frequency oscillations in the hippocampus. The spiking latency following synaptic input was shorter for only a narrow window between 30–60 degrees (Figure 4A). It should be noted here that in experiments with fast gamma injection several synaptic inputs arrive within a millisecond of each other. In such cases, it is difficult to distinguish between the two inputs; however even when synaptic inputs arrive at distinct time points we still do not find any significant response tuning. We find that neither slow nor fast gamma oscillations exhibit strong phase coding.

Network oscillations provide a broad temporal framework reflecting the general behavior of the animal, for instance, theta oscillations during locomotion or gamma oscillations during sensory stimulation [5], [14], [40]. Within this framework is embedded a second level of precise information encoded in the form of phase coding in neurons. Phase coding is a possible method to amplify signal to noise, by eliminating weak inputs [40], [42]. Our findings highlight the fact that neuronal response is modulated with respect to the phase of the afferent inputs as well as the frequency of the network oscillations in an analog manner (Figure 4B). The output readout of a hippocampal neuron thus integrates two input features – frequency of network activity and timing of synaptic inputs. The frequency of network activity is characteristic of the behavioral context and the timing of synaptic inputs is a possible correlate of the sensory information.

### Network vs. intracellular computation

Membrane potential oscillations *in vivo* have been hypothesized to be caused by interactions between dendritic excitation and somatic inhibition. Thus the neuron constantly gets a barrage of synchronous synaptic inputs which interact with asynchronous input at various input phases. While network input differs from our intracellular input in having a dendritic rather than somatic location, we consider this a reasonable approximation on four grounds [4]. First, we find that summed synaptic input from multiple input electrodes behaves in a similar manner to single-electrode synaptic input, suggesting that our results are not very sensitive to the extent of dendritic activation (Figure 5). Extensive literature on summation in CA1 neurons demonstrate that the nature of inputs decide the summation properties. Non-linear summation is seen typically when inputs are spatially or temporally clustered [36], [43]. Additionally, the summation properties also depend on the readout parameter; in our experiments we have used EPSP area which is a temporally broad but robust signal. Given the nature of our input and readout parameter, we expect summation of be sub-linear. Saturation of inputs will result in saturation of EPSP area, which we do not observe. Second, we obtain comparable phase dependence of our intracellular EPSPs to extracellularly recorded field EPSPs from *in vivo* studies in which dendritic network input is present [37]. In this earlier study, Wyble et. al. describe findings of field EPSP slope dependence on theta phase. They report the slope to be largest ∼290 degrees and smallest ∼100 degrees of intracellular theta. Our intracellularly recorded data are in agreement with this (Figures 4C). Third, resonance in membrane potential responses was seen at theta frequency in slice experiments in hippocampal CA1 neurons using sinusoidal current injection [30]. This resonance frequency matched the spontaneous oscillation frequency suggesting the same voltage dependent phenomenon driven by sinusoidal injection as seen *in vivo*. Fourth, we were able to replicate the phase-dependence of our intracellular EPSPs in a detailed compartmental model. This model behaved in approximately the same manner for somatic current injection as for spatially separated theta-frequency inhibitory and excitatory synaptic input (Figure 4D). Together, these observations suggest that our approach of simulating network activity using sinusoidal intracellular current injection is a reasonable approximation to actual periodic network activity. Furthermore, the simulations suggest that the outcome of such stimuli are relatively insensitive to variations within the theta frequency range (4–12 Hz) and also to overlaying gamma frequencies on the theta rhythm. Using intracellular recordings, we are able to extend the EPSP phase-dependence analysis to show a significant increase in probability of AP generation at shorter latencies between phases of 0–180 degrees and a significant reduction in probability at the hyperpolarizing phases between 270–360 degrees. For example, this could enhance sensitivity of the cell to smaller inputs in this phase range (Figure 4A). We interpret the anti-correlation between spiking activity and EPSP area as arising from the K-channel activation mediating post-spike hyperpolarisation. This phenomenon of increasing duration of hyperpolarisation has also been shown to enhance the precision of the next AP [44].

### Computational implications of robust summation during theta

The hippocampus has been extensively studied because of its role in short-term memory [45]. The hippocampus temporarily binds together information from various sensory modalities to represent the information holistically [5], [46]–[48]. Sensory input from various cortical areas impinges on the hippocampus in the form of afferent inputs while their membrane potential oscillates at various frequencies. Since the response is phase-dependent, there is only a narrow time window in which the neuron can maximally transmit and thus receive information. This has led to the hypothesis that oscillations are a mechanism to synchronize neurons and play a critical role in input integration [5], [49]–[51].

It has been suggested that the integration of multiple sensory inputs into a single percept (poly-modal binding) can be broken down into two distinct inputs. One, temporally synchronous sensory inputs sent to the hippocampus from the various cortical regions and two, phase-locking oscillations between the hippocampal-cortical circuits [5], [40], [46], [50], [52]. In this model, the hippocampus integrates both these inputs and records a unified ‘memory engram’ of the event. The present study suggests a mechanism for such integration. Our findings show that the hippocampus preferentially transmits summed inputs arriving in a narrow phase window of theta frequency current injection (Figure 6B). This provides the network a ‘window of opportunity’ where the neuron's response is selectively amplified. Neurons in the brain constantly encounter inputs from a large number of sources which reflects in the network activity. It still remains highly debated whether the oscillations are essential to the neuronal function, or whether the neuronal functions are carried out despite the fluctuations. Our results show that although the neuronal response oscillates with the background input, a functionally critical property - summation gain - remains robust to oscillatory background inputs (Figure 7). Summation remains unperturbed even as background input fluctuates between intense and sparse activity. This is a theoretically desirable property for a distributed system like the brain with large input convergence and large output divergence.

## Materials and Methods

All of the experimental procedures were approved by the National Centre for Biological Sciences institutional animal ethics committee, in accordance with the guidelines of the Government of India.

### Electrophysiology

400 µm transverse hippocampal slices were prepared from 4 to 6 week old male Wistar rats using a vibratory microtome (Vibratome 1000 classic series, Vibratome, USA) in ice-cold artificial cerebro-spinal fluid (aCSF) containing (in mM)—118 NaCl, 2.5 KCl, 2.5 CaCl_2_, 1.25 Mg Cl_2_, 1.25 NaH_2_PO_4_, 26 NaHCO_3_, and 10 glucose, saturated with carbogen gas (95% O_2_, 5% CO_2_). Slices were equilibrated in aCSF at room temperature (22–25°C) for 60 min. The response was recorded using whole cell recordings in current clamp mode. Internal solution used to fill the pipette contained (in mM) – 120 K-gluconate, 6.66 KCl, 10 HEPES, 2 MgCl_2_, 0.3 Na-GTP, 0.2 EGTA, 4 Mg-ATP, 14 Phosphocreatine and was adjusted with KOH to pH 7.2–7.3 and with sucrose to osmolarity 280–300 mOsm. Whole cell voltage recordings from the CA1 soma were recorded with an amplifier (HEKA EPC10) under visual guidance by infrared differential interference contrast video microscopy. Patch pipettes were pulled from standard-wall borosilicate tubing. Junction potential and capacitance were corrected for. Experiments in which the series resistance or input resistance changed by more than 30% were discarded and only cells with resting membrane potential negative to −55 mV were included in the analysis. Voltages were not corrected for liquid junction potential, estimated to be ∼7 mV. Signals were filtered at 5 k Hz and analysed offline.

### Input Design

#### Tonic Sinusoidal Current Injection

We first recorded the neuronal response to tonic sinusoidal current injection at increasing amplitudes ranging from 50–500 pA in steps of 50 pA. The patched neurons were held at resting potential. Based on these recordings we fixed 4 Hz, 40 Hz and 100 Hz sinusoidal wave at a peak-to-peak amplitude 60 pA and zero mean as our reference sinusoidal inputs (Figure 1). This generated ∼5–10 mV and ∼2–5 mV peak to peak membrane potentials for theta and gamma respectively, which is in the range observed *in vivo* [3], [28], [33]. The cell did not spike with the background sinusoidal current injection.

The sinusoidal injected current modeled intracellular membrane potential fluctuations caused by rhythmic network activity. One of the widely accepted *ex vivo* models of theta activity in the hippocampus is the somato-dendritic interference model [27]. This model replicates many characteristic features of the extracellular and intracellular membrane potential changes recorded in an animal running in a track [2]. According to this model, the phasic excitatory input impinges onto the dendrite and phasic inhibitory input impinges proximal to the soma. Our sinusoidal current injection is an attempt to simplify this model further. All oscillatory effects are restricted at a single point since our readouts are only intra-cellular. We validate this protocol further using a simulated model of CA1 neuron.

In the case of gamma rhythm in CA1 neurons, the oscillation frequency predominantly depends on GABA receptor mediated inhibition. The slow and fast gamma rhythms are routed through CA3 and medial EC, which synapse onto the fast-spiking basket cells whose firing drives gamma oscillations [53]. Since both slow and fast gamma rhythms are routed through GABAergic interneurons, we approximate the intrinsic effects by sinusoidal current injections at 40 and 100 Hz. Additionally, since we use only impulse afferent inputs the interaction caused by the coherence in fluctuations between the CA3 and CA1 neurons can be safely neglected.

#### Calibration of Afferent Input Summation

We then calibrated the summation properties of hippocampal CA1 responses to near threshold afferent inputs without somatic current injection. The stimulating electrodes used to inject synaptic input consisted of an array of custom made twisted bipolar electrodes (Nichrome, 50 µm outer diameter). The electrodes were aligned as close together as possible and placed along the dendritic axis stimulating the Schaffer Collaterals a few millimeters from the recorded neurons. In previous work we have shown that this arrangement results in a very small overlap between electrodes when tested using cross-electrode paired pulse facilitation [54]. The synaptic inputs are distributed along the dendrite and largely stimulate the Stratum Radiatum layers (Methods S1 and Figure S5). The input currents ranged between 0.04–0.3 mA. Currents were delivered using a Master-8 (A.M.P.I.). We delivered a single pulse of current (duration 60 µs) synchronously to the Schaffer Collaterals as the afferent input. We used 4 input electrodes and were thus able to give 15 stimulus combinations (2^Nel^-1), of which 11 were summed combinations (N = 12 cells) (Figure 5). We also injected noisy background current into the soma while giving synaptic input patterns (3 patterns with 2 electrodes) at the Schaffer Collaterals. The currents injected were Gaussian noise (mean ± sd, 0±20 pA and 0±40 pA). The background input given for 40 ms and the afferent inputs were given at the onset of the background stimuli. This protocol did not elicit multiple APs.

#### Combining sinusoidal current injection with afferent inputs

In an independent set of recordings we combined sinusoidal current injection with afferent inputs (N = 13 neurons). The patched neurons were held at resting potential. The sinusoidal current injection was delivered at the same 60 pA amplitude and zero mean (N = 20 cells). Synaptic inputs were overlaid with the current injection to the cell at different phase values with respect to the sinusoidal current injected at the soma. We refer to the phase of synaptic input with respect to the background sinusoidal input as the “input phase”. Synaptic input was delivered at 16 input phase values of – 30, 45, 60, 90, 120, 135, 150, 180, 210, 225, 240, 270, 300, 315, 330 and 360 degrees. Thus if the synaptic input coincided with the start of the sinusoid, the input phase was zero, and if the synaptic input was a quarter cycle later the input phase was 90 degrees (Figure 2A). Input patterns were interleaved between single, multiple synaptic inputs and 16 input phase values with 5 repetitions at each input pattern. We also recorded responses to synaptic inputs without background sinusoidal injection as a control measure. Our response readouts were spiking probability, latency, phase of AP firing and area under the EPSP with respect to the sinusoidal current. Spiking probability was calculated as the ratio of number of trials in which the cell spiked to the total trials with data pooled from all cells for a given phase and frequency value. This gives us a single probability for each phase value. The spiking probability at each input was normalized to control spiking probability without background input to get the relative spiking probability. The statistics were calculated using a Binomial Test which gives the significance of the deviation of relative spiking probabilities from 1 (control). Latency was defined as the time of spiking from the stimulus artifact measured in ms. Latencies were compared only for cells which spiked at least once at every phase and frequency value. Phase of AP firing was calculated as the phase of output spike with reference to the input sinusoidal current injection. EPSP area was calculated over a time period 200 ms.

### Model

We modelled inputs to the CA1 neuron using the GENESIS simulator using a 50 µs time step [55]. CA1 neurons were modeled as 25-compartment neurons modified from Bhalla (2011) [56] which in turn was based on Traub et al. [57] with the inclusion of NMDA, AMPA and GABA receptors. Model geometry and channel details are presented in Table S1. In brief, GABA inputs were clustered on the soma and on the most proximal apical and basal compartments (Figure 3). Glutamate and NMDA receptors were distributed throughout the apical dendrite including its branches. Synaptic weights were set up using a Gaussian-based distributions (Mean synaptic probability of 0.4). We designed 4 simulation experiments using this model.

1. We first recorded the CA1 neuron's response to tonic sinusoidal current injection at increasing amplitudes ranging from 50–500 pA in steps of 50 pA. This generated ∼5–10 mV peak to peak membrane potentials for theta frequency injection similar to the experimental results.
2. Network activity simulated using Current Injection – We replicated our experiment here. We gave synchronous synaptic inputs overlaid with sinsusoidal somatic current injection to the CA1 neuron at 16 different phase values with respect to the sinusoidal wave (Figure 3A).
3. Network activity simulated using Synaptic Input patterns – We replicated the somato-dendritic interference model here. Excitatory and inhibitory pre-synaptic inputs were given to the CA1 in order to generate a 4 Hz oscillatory sine wave at the soma. Excitatory inputs were delivered at the apical dendrites and their branches, whereas inhibitory inputs were present only at the soma and proximal basal dendrites, and the proximal 120 µm of the apical dendrite [4] (Figure 3B). The pre-synaptic input was modelled as input spikes that arrived at each synapse as Poisson spike trains with a probability modulated with a sine wave. The positive part of the sine wave modulated the glutamate/NMDAR inputs, and the negative part of the sine wave was rectified and used to modulate the probability of inputs to the GABA receptors. Synaptic weights were calibrated to generate a reasonable sine-wave potential at the soma (Figure 3Bii). In addition to the sinusoidal current or synaptic input, synchronous excitatory inputs were overlaid to represent our electrode input to the Schaffer Collaterals. Model voltage changes were recorded at the soma (Figures 3A and 3B subpanel iii).
4. Theta-gamma coupled model – We designed 2 simulation experiments with cross-coupled frequency input. The power and timing of gamma rhythm on theta are the two key parameters of cross-frequency coupled inputs in hippocampus [20], [21]. The power of gamma frequency was gradually modulated in time. We designed 2 cases – where the maximal power gamma input was either incorporated at 90 degrees or at 270 degrees (Figure 4E) [20], [21]. We designed these input stimuli with both current injection and synaptic input pattern protocols. As in model 3, we provided excitatory (GluR and NMDAR) synaptic input in the dendrites, and inhibitory (GABAR) synaptic input in the soma and near-proximal dendrites.

In addition to these four simulated experiments, we also used the model to investigate the contribution of voltage gated channels, and specifically K^+^ channels, to the phase dependence of EPSP area. To do this we computed the EPSC (excitatory post-synaptic current) due to the same simulated volley synaptic input as in the ‘experiments’ above. We then computed the sum of all the somatic voltage-gated ion channel conductances, as well as the sum of all the somatic K^+^ channel conductances, at a series of time-points from 5 to 35 ms. We did this for all the stimulus-phase combinations in ‘experiment’ 2 above. We also computed the input resistance of the model neuron at rest. For each of the sampled time-points we computed EPSP = EPSC/total conductance, where the total conductance was 1/input resistance+ion-channel conductance. We weighted the sampled time-points by their spacing, and summed them up. The net effect of this procedure was to compute the convolution of the EPSC with the instantaneous resistance of the cell. We carried out this convolution for every stimulus-phase, and repeated the calculations with just the K^+^ channel conductances. These calculations were done using Microsoft Excel.

All analysis except the EPSC convolution ones was done using MatlabR2007. All numerical data are reported as means ± SEM.

## Supporting Information

Figure S1 (**A**) The latency of the first spike is inversely proportional to the current amplitude. This effect is most prominent in the theta frequency (4 Hz) current injection. (**B**) Phase delay is also inversely proportional to the current amplitude but only in the case of theta frequency current injection. In the cases of slow and fast gamma frequencies, the phase shift is independent of the current amplitude.
(PDF)

Figure S2 (**A**) Plot shows the output spike lag as a function of input phase. The coefficient of variation of phase lag is 0.12, 0.11 and 0.10 for theta, slow gamma and fast gamma frequencies. (**B**) Plot shows the dynamically changing relationship between the EPSP area and probability of spiking as the phase of the afferent input changes.
(PDF)

Figure S3 (**A–B**) Somatic current injection and network activity generated using a barrage of excitatory and inhibitory inputs at 8 Hz and 12 Hz caused similar phasic modulation as seen with the 4 Hz background. However, the phase shift between the current injection and synaptic input backgrounds increased with the increase in the input frequency. (**C**) The simulations with somatic current injection and synaptic input generated background were run with sub-threshold afferent inputs (cell did not spike). The phase tuning was low and negatively correlated with the phasic tuning seen with spiking inputs. This suggests the small effect of driving force changes caused by the background on phase tuning. (**D**) EPSP area modulation by voltage-gated ion conductances. The modulation phase is similar to that of the experimental and simulated EPSP area (Figure 4). The K^+^ channels on their own produce almost the same amount of modulation as all the ion channels.
(PDF)

Figure S4 (**A**) Theta-gamma coupled inputs paired with afferent inputs were simulated using patterned barrage of excitatory and inhibitory inputs. Two types of input were given – one, with the maximal power of gamma frequency input aligned with the depolarized phase of theta input (above). Two, the maximal power of gamma input coincided with the hyperpolarized phase of theta input (below). (**B**) Response tuning with gamma on theta depolarizing case correlated positively with the response tuning seen with just theta background. However, no response tuning was seen with gamma during hyperpolarizing theta.
(PDF)

Figure S5 (**A**) Fluorescence image of a CA1 neuron sparsely loaded (ballistically) with Calcium-green1 dextrans (40×, Scale Bar 10 mm, left). We measured fluorescence changes in the primary branches to check whether the inputs were clustered or distributed. To get high resolution and high-speed movies (123 Hz using the same CCD camera) we imaged a small region around the branch point. Traces of ΔF/F in the ROIs (white dashed line) indicated the distribution of inputs across the dendritic branches. (**B**) Inputs from CA3 axons were distributed on multiple branches of the CA1 dendrites in 8 out of 9 cells imaged.
(PDF)

Figure S6 (**A**) Latency of evoked APs by summed inputs (dark colors) with single electrode inputs (light colors) for the same group of cells shows similar tuning but smaller absolute values in the case of summed inputs.
(PDF)

Methods S1 **The distribution of CA1 synapses receiving input on the dendrite was investigated using calcium dye-loading technique.** This section presents the details of the experiments conducted.
(PDF)

Table S1 **Table contains CA1 neuron model geometry and channel details.** Inputs to the CA1 neuron were modelled using the GENESIS simulator.
(PDF)
