# Supplementary figures and images for: Theta Frequency Background Tunes Transmission but Not Summation of Spiking Responses
Source: PLoS One. 2013 Jan 31;8(1):e55607. doi: 10.1371/journal.pone.0055607 (PMC3561309; doi:10.1371/journal.pone.0055607)

Figure S1

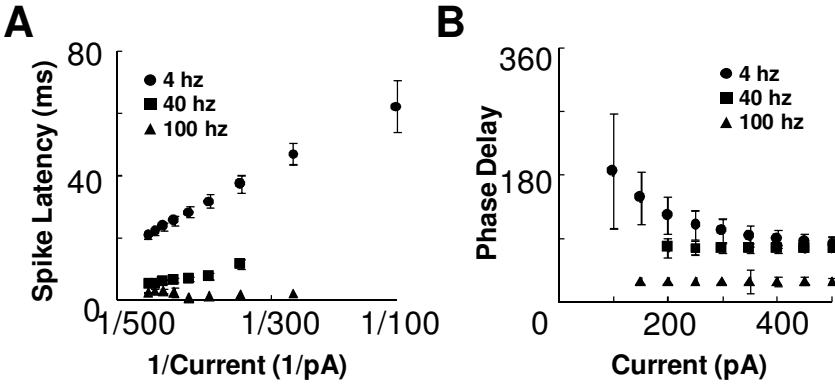

Supplement: Figure S1 — (A) The latency of the first spike is inversely proportional to the current amplitude. This effect is most prominent in the theta frequency (4 Hz) current injection. (B) Phase delay is also inversely proportional to the current amplitude but only in the case of theta frequency current injection. In the cases of slow and fast gamma frequencies, the phase shift is independent of the current amplitude. (PDF) [file pone.0055607.s001.pdf]

**Figure S2**

● Theta      ● Slow Gamma      ● Fast Gamma

**A**

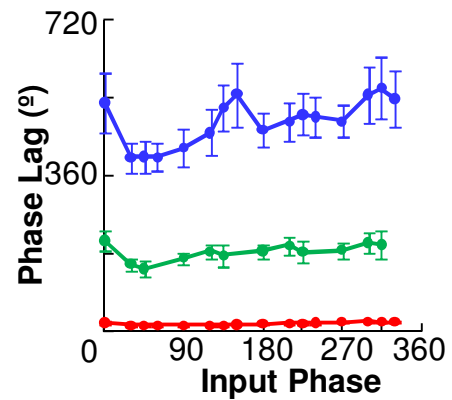

**B**

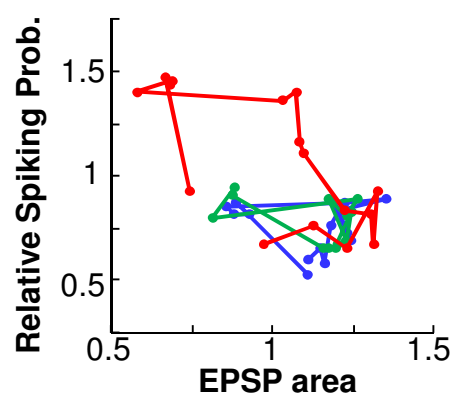

Supplement: Figure S2 — (A) Plot shows the output spike lag as a function of input phase. The coefficient of variation of phase lag is 0.12, 0.11 and 0.10 for theta, slow gamma and fast gamma frequencies. (B) Plot shows the dynamically changing relationship between the EPSP area and probability of spiking as the phase of the afferent input changes. (PDF) [file pone.0055607.s002.pdf]

Figure S3

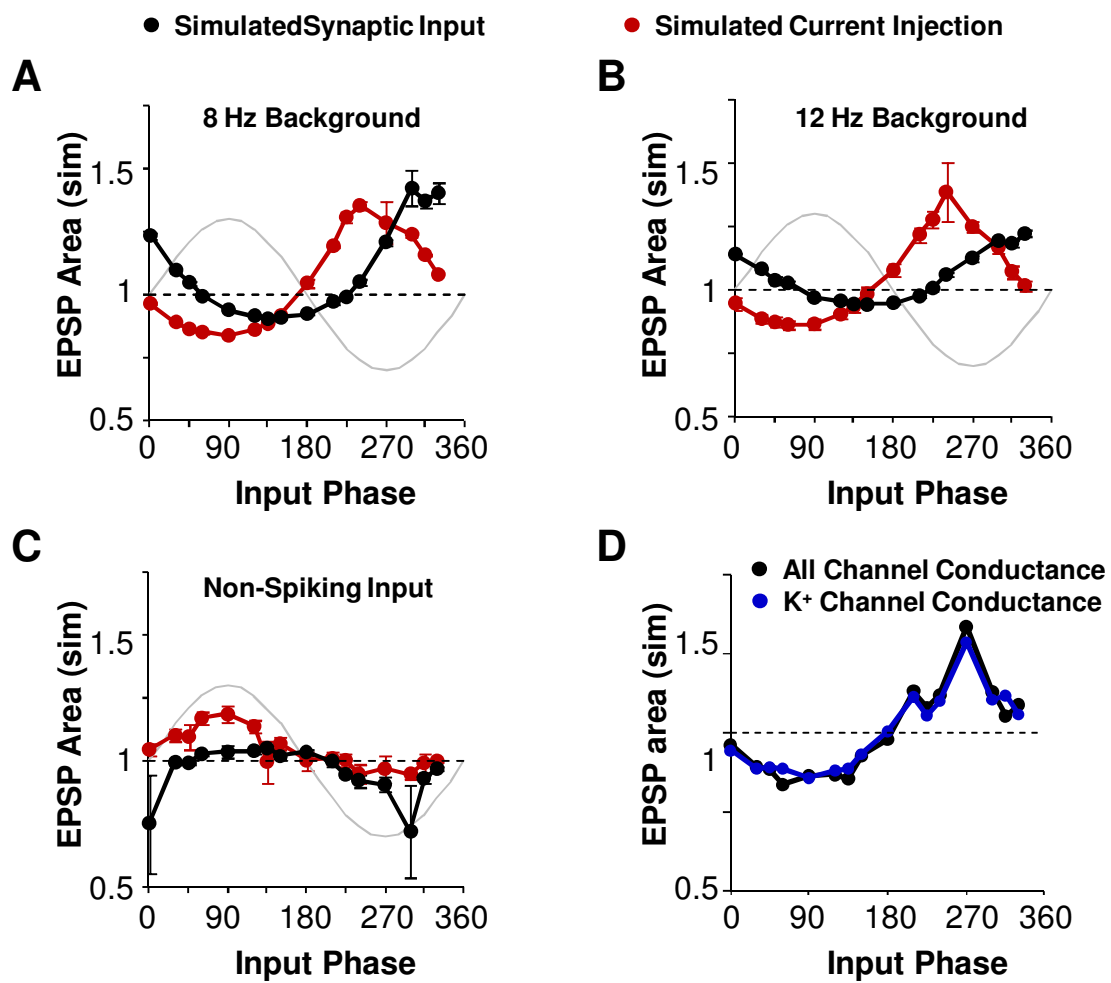

Supplement: Figure S3 — (A–B) Somatic current injection and network activity generated using a barrage of excitatory and inhibitory inputs at 8 Hz and 12 Hz caused similar phasic modulation as seen with the 4 Hz background. However, the phase shift between the current injection and synaptic input backgrounds increased with the increase in the input frequency. (C) The simulations with somatic current injection and synaptic input generated background were run with sub-threshold afferent inputs (cell did not spike). The phase tuning was low and negatively correlated with the phasic tuning seen with spiking inputs. This suggests the small effect of driving force changes caused by the background on phase tuning. (D) EPSP area modulation by voltage-gated ion conductances. The modulation phase is similar to that of the experimental and simulated EPSP area (Figure 4). The K+ channels on their own produce almost the same amount of modulation as all the ion channels. (PDF) [file pone.0055607.s003.pdf]

Figure S4

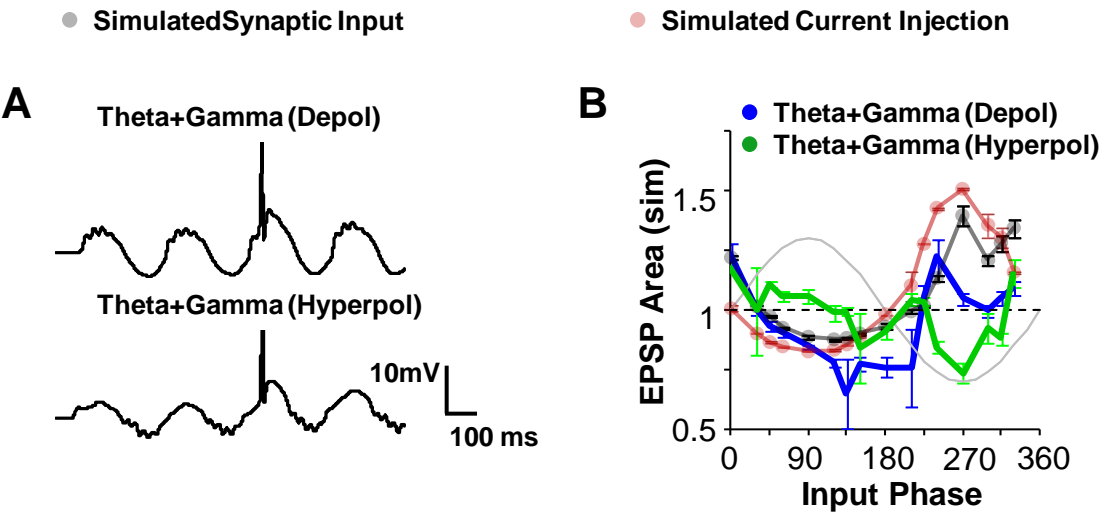

Supplement: Figure S4 — (A) Theta-gamma coupled inputs paired with afferent inputs were simulated using patterned barrage of excitatory and inhibitory inputs. Two types of input were given – one, with the maximal power of gamma frequency input aligned with the depolarized phase of theta input (above). Two, the maximal power of gamma input coincided with the hyperpolarized phase of theta input (below). (B) Response tuning with gamma on theta depolarizing case correlated positively with the response tuning seen with just theta background. However, no response tuning was seen with gamma during hyperpolarizing theta. (PDF) [file pone.0055607.s004.pdf]

**Figure S5**

**A**

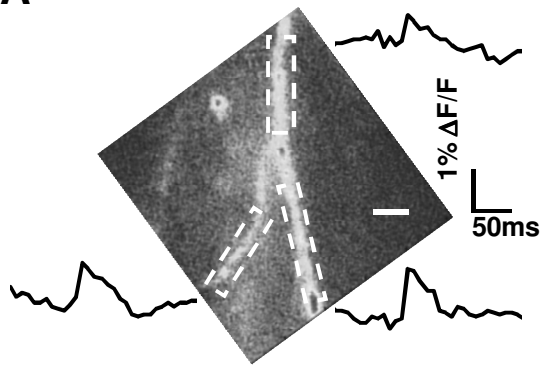

**B**

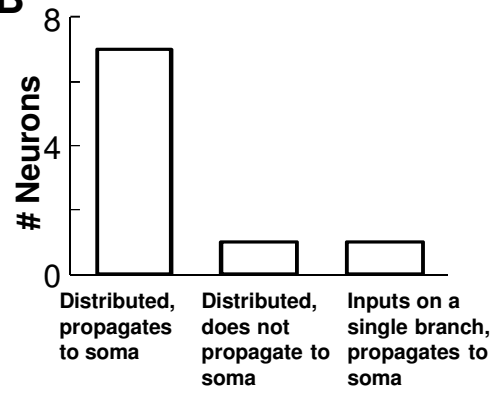

Supplement: Figure S5 — (A) Fluorescence image of a CA1 neuron sparsely loaded (ballistically) with Calcium-green1 dextrans (40×, Scale Bar 10 mm, left). We measured fluorescence changes in the primary branches to check whether the inputs were clustered or distributed. To get high resolution and high-speed movies (123 Hz using the same CCD camera) we imaged a small region around the branch point. Traces of ΔF/F in the ROIs (white dashed line) indicated the distribution of inputs across the dendritic branches. (B) Inputs from CA3 axons were distributed on multiple branches of the CA1 dendrites in 8 out of 9 cells imaged. (PDF) [file pone.0055607.s005.pdf]

Figure S6

Multiple Input : ● Theta ● Slow Gamma ● Fast Gamma  
Single Input : ● Theta ● Slow Gamma ● Fast Gamma

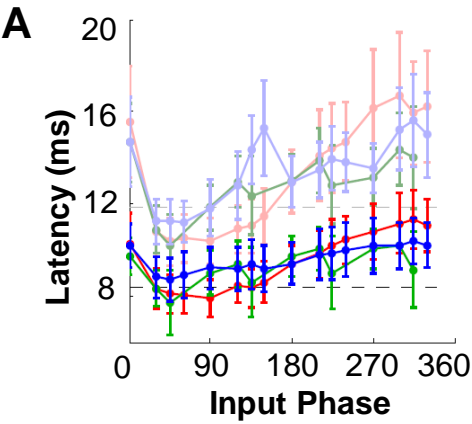

Supplement: Figure S6 — (A) Latency of evoked APs by summed inputs (dark colors) with single electrode inputs (light colors) for the same group of cells shows similar tuning but smaller absolute values in the case of summed inputs. (PDF) [file pone.0055607.s006.pdf]
