# Supplementary material for: Theta Frequency Background Tunes Transmission but Not Summation of Spiking Responses
Source: PLoS One. 2013 Jan 31;8(1):e55607. doi: 10.1371/journal.pone.0055607 (PMC3561309; doi:10.1371/journal.pone.0055607)
Supplement: Methods S1 — The distribution of CA1 synapses receiving input on the dendrite was investigated using calcium dye-loading technique. This section presents the details of the experiments conducted. (PDF) [file pone.0055607.s007.pdf]

Supporting Methods : Theta frequency background tunes transmission but not summation of spiking responses

Distributed Synaptic Inputs with Synchronous Inputs

Previous studies [1,2] have shown that the distribution of synapses receiving input on the dendrite decide the mode of integration in CA1 neurons. To investigate the distribution of input synapses on the dendrite we loaded the slices using ballistic delivery of fluorescent dye. Calcium green-1 dextrans conjugated dye (Molecular Probes C-6765) was coated on gold particles (1-1.5 µm radius, Aldrich 326585) and delivered into the slice preparation with a 'gene-gun'. This method results in loading of individual cells contacted by these particles [3]. Metal filters were used to protect the tissue from shock wave generated by the gun at high pressure (60-80 psi). An Olympus microscope (IX 50) with fluorescence attachment was used to image the labeled structures. Videos were captured on a high speed cooled CCD camera (Andor DV iXON 887 BI) at 122 Hz. Regions were selected where only a single neuron was loaded with discernable dendritic segments (N = 9 cells). In 4 out of 9 cells we imaged the primary, secondary and tertiary dendrites. In the remaining 5 cells we imaged the primary and secondary dendrites.

We selected regions of interest in the digitized image, where a single neuron was loaded. We delivered inputs using our electrode array and analyzed which of the imaged dendritic branches responded. We found that even inputs from a single electrode (presumably stimulating a bundle of nearby axons) impinge on multiple branches on the dendrite (in N=8 out 9 slices, Figure S5). Thus we conclude that the summations investigated in this study represented spatially distributed, synchronous synaptic stimuli.

1. Cash S, Yuste R (1999) Linear summation of excitatory inputs by CA1 pyramidal neurons. Neuron 22: 383-394.
2. Gasparini S, Magee JC (2006) State-dependent dendritic computation in hippocampal CA1

pyramidal neurons. J Neurosci 26: 2088-2100.
3. Kettunen P, Demas J, Lohmann C, Kasthuri N, Gong Y, et al. (2002) Imaging calcium dynamics in the nervous system by means of ballistic delivery of indicators. J Neurosci Methods 119: 37-43.
