# Supplementary material for: Theta Frequency Background Tunes Transmission but Not Summation of Spiking Responses
Source: PLoS One. 2013 Jan 31;8(1):e55607. doi: 10.1371/journal.pone.0055607 (PMC3561309; doi:10.1371/journal.pone.0055607)
Supplement: Table S1 — Table contains CA1 neuron model geometry and channel details. Inputs to the CA1 neuron were modelled using the GENESIS simulator. (PDF) [file pone.0055607.s008.pdf]

Biophysical CA1 model constants

All voltages are in V.					
EREST	-0.06				
ENa	0.055				
EK	-0.075				
ECa	0.08				

Membrane parameters:

RM ($ohm.m^2$)	1
RA (ohm.m)	1
CM (F/m^2)	0.03

We use the Hodgkin-Huxley form for gating
of a voltage-gated channel:
g = gmax * m^x * h^y
where m and h are gating variables, and x and y are powers.
m and h obey differential equations of the form:
dm/dt = m_alpha * (1 - m) - m_beta * m
In the equations below, this differential form is assumed for
all gates unless stated otherwise.

Voltage-gated Ca channel

g = gmax * s^2 * r	A	B	C	D	F
s_alpha = (A + B * x) / (C + exp((x + D) / F))	1600	0	1	-0.005	-0.01389
s_beta = (A + B * x) / (C + exp((x + D) / F))	178	2.00E+04	-1	-0.0089	0.005

r_alpha = 5 * exp(-50 * (V + 0.06)) for V > -0.06
r_alpha = 5 for V < -0.06

Ca concentration pool (note that units are NOT uM)
d(conc)/dt = sum(Ca_influx) - conc / 0.01333 in main cell
d(conc)/dt = sum(Ca_influx) - conc / 0.004 in spines

K_AHP

g = gmax * z
z_alpha = 0.02 * conc   for conc < 250
z_alpha = 10.0   for conc >= 250

z_beta = 1.0 + 0.02 * conc   for conc < 250
z_beta = 1.0 + 10.0   for conc >= 250

K_C

g = gmax * x * z
for V < -0.01:
x_alpha = exp (53.872*(V - EREST) - 0.66835)/0.018975
x_beta = 2000*exp((EREST + 0.0065 - V)/0.027) - x_alpha(V)
for V > -0.01:
x_alpha = 2000*exp((EREST + 0.0065 - V)/0.027)
x_beta = 0

The z gate is computed directly from Ca concentration, not
using a differential equation:
For Ca < 125:
z = Ca * 0.008
For Ca > 125
z = 1

Na

g = gmax * m^2 * h	A	B	C	D	F
m_alpha = (A + B * x) / (C + exp((x + D) / F))	-15008	3.20E+05	-1	0.0469	-0.004
m_beta = (A + B * x) / (C + exp((x + D) / F))	5572	2.80E+05	-1	0.0199	0.005
h_alpha = (A + B * x) / (C + exp((x + D) / F))	128	0	0	0.043	0.018
h_beta = (A + B * x) / (C + exp((x + D) / F))	4000	0	1	0.02	-0.005

K_DR

g = gmax * n	A	B	C	D	F
m_alpha = (A + B * x) / (C + exp((x + D) / F))	-398.4	-1.60E+04	-1	0.0249	-0.005
m_beta = (A + B * x) / (C + exp((x + D) / F))	250	0	0	0.04	0.04

K_A

g = gmax * phosph_scaling * n * q	A	B	C	D	F
n_alpha = (A + B * x) / (C + exp((x + D) / F))	-938	-2.00E+04	-1	0.0469	-0.01
n_beta = (A + B * x) / (C + exp((x + D) / F))	348.25	1.75E+04	-1	0.0199	0.01
q_alpha = (A + B * x) / (C + exp((x + D) / F))	1.6	0	0	0.073	0.018
q_beta = (A + B * x) / (C + exp((x + D) / F))	50	0	1	0.0499	-0.005

phosph_scaling goes from 1 to 0.25 as p-ERKII increases.
phosph_scaling = (1 + 3 * exp (-pERKII / 1.5)) / 4
p-ERKII is assumed to have a ceiling of 5. Although the initial

amount of ERKII is 3.6 uM, diffusion may lead to higher p-ERKII.

GluR channel

	$\tau 1$	$\tau 1$
$g_{syn}(t) = (A.g_{max}./(\tau 1 - \tau 2)).(\exp(-t/\tau 1) - \exp(-t/\tau 2))$	2 msec	9 msec

A is a normalization constant obtained as follows:

$\tau pk = \tau 1 * \tau 2 * \ln(\tau 1 / \tau 2) / (\tau 1 - \tau 2)$

$A = (\tau 1 - \tau 2) / (\tau 1 * \tau 2 * \exp(-\tau pk / \tau 1) - \exp(-\tau pk / \tau 2))$

GABA channel

$g_{syn}(t) = g_{max}./(\tau 1 - \tau 2)).(\exp(-t/\tau 1) - \exp(-t/\tau 2))$	15 msec	15 msec

NMDAR channel: conductance term

$g_{syn}(t) = Block.(A.g_{max}./(\tau 1 - \tau 2)).(\exp(-t/\tau 1) - \exp(-t/\tau 2))$	20 msec	20 msec	
NMDAR channel: Mg block term			[Mg]
	eta	gamma	1.2
Block = 1/eta * (1/eta - [Mg]*exp(-V * gamma))	0.28	62	

NMDAR channel: Ca influx term. This is computed separately from the conductance term using identical equations but using Eca = 0.1 V which differs from the current reversal potential. We assume that Ca carries 1/10 of the NMDA channel conductance.

**Cell model compartmental structure**			Ca	K_AHP	K_C	Na	K_DR	K_A	AMPAR	NMDAR
Compartment name	Length (um)	Dia (um)	0	0	0	0	0	0	0	0
Apical_19	120	2.6	70	8	50	40	60	100	700	200
Apical_18	120	2.6	70	8	50	40	60	100	700	200
Apical_17	120	2.6	70	8	50	40	60	100	700	200
Apical_16	120	2.6	70	8	50	40	60	100	700	200
Apical_15	120	2.6	70	8	50	40	60	100	700	200
Apical_14	120	2.6	70	8	50	50	60	100	700	200
Apical_13	120	2.6	70	8	50	60	60	100	700	200
Apical_12	120	3	70	8	50	80	60	100	700	200
Apical_11	120	3	80	8	200	150	100	0	700	200
Apical_10	120	4	40	8	100	300	250	50		
Soma	125	8.46	80	8	200	150	100			
Basal_8	110	3.84	120	8	100	200	200			
Basal_6	220	3.84	70	8	50					
Basal_5	220	3.84	50	8	50					
Basal_3	220	3.84	70	8	50	80	60	100	700	200
Lat_11_1 (Attached to apical_10)	85	2	70	8	50	40	60	100	700	200
lat_11_2	85	1.8	70	8	50	40	60	100	700	200
lat_11_3	80	1.5	70	8	50	40	60	100	700	200
lat_11_4	80	1.5	70	8	50	40	60	100	700	200
lat_13_1 (Attached to apical_12)	57	1	70	8	50	40	60	100	700	200
lat_13_2	57	1	70	8	50	40	60	100	700	200
lat_14_1	10	1	70	8	50	40	60	100	700	200
lat_14_2	10	1	70	8	50	40	60	100	700	200
lat_14_3	70	1	70	8	50	40	60	100	700	200
lat_14_10	30	1	70	8	50	40	60	100	700	200
lat_15_1	60	1	70	8	50	40	60	100	700	200
lat_15_2	60	1								
spine_neck_14_1 (attached to lat_14_1)	0.5	0.1	60	0	0	0	0	0	700	200*
spine_head_14_1	0.5	0.5								
spine_neck_14_2 (attached to lat_14_2)	0.5	0.1	60	0	0	0	0	0	700	200*
spine_head_14_2	0.5	0.5								
spine_neck_14_3 (attached to lat_14_10)	0.5	0.1	60	0	0	0	0	0	700	200*
spine_head_14_3	0.5	0.5								

* 10% of the NMDA current was assumed to be carried by Ca2+ ions. This was implemented as a separate NMDA receptor entity in the spine heads, with identical kinetics but connecting only to the Ca2+ pool
